# Association between neutrophil-to-lymphocyte ratio and the prognosis of patients with rheumatoid arthritis: a meta-analysis

**DOI:** 10.3389/fimmu.2026.1770565

**Published:** 2026-04-01

**Authors:** Yi Dong, Yu Zhong, Huahong Zhou, Dong Cheng

**Affiliations:** 1 Department of Rheumatology and Nephrology Integrating Traditional Chinese and Western Medicine, Jinhua Traditional Chinese Medicine Hospital Affiliated to Zhejiang Chinese Medical University, Jinhua, Zhejiang, China; 2 Department of Rheumatology and Nephrology Integrating Traditional Chinese and Western Medicine, Tongde Hospital of Zhejiang Province, Hangzhou, Zhejiang, China

**Keywords:** meta-analysis, neutrophil-to-lymphocyte ratio, NLR, rheumatoid arthritis, systematic review

## Abstract

**Background:**

Rheumatoid arthritis (RA) is an autoimmune disease characterized by chronic systemic inflammation, posing a high risk of death, particularly from cardiovascular events. Finding simple and cost-effective prognostic biomarkers is crucial for risk stratification and improved patient management. The neutrophil-to-lymphocyte ratio (NLR), as a systemic inflammatory marker, has shown prognostic value in various diseases, but its comprehensive evidence in RA remains unclear.

**Methods:**

Following the PRISMA 2020 guidelines, relevant literature up to October 2025 was systematically searched in PubMed, Embase, Web of Science, and Cochrane databases. Observational studies were included. Random-effects models were used to pool odds ratios (OR) and 95% confidence intervals (CI). Robustness and publication bias were assessed using heterogeneity tests (I²), sensitivity analyses, and Egger’s tests. Evidence quality was graded using the GRADE system.

**Results:**

Overall, seven studies were analyzed, and the meta-analytic findings indicated that elevated NLR was significantly correlated with all-cause mortality (OR = 1.70, 95%CI: 1.39-2.09, P<0.00001) and cardiovascular mortality (OR = 2.60, 95%CI: 1.61-4.21, P = 0.0001) in RA patients, and also with reduced disease remission rate (OR = 0.81, 95%CI: 0.68-0.96, P = 0.02). Heterogeneity for all outcomes was low (I²=0-16%), sensitivity analysis confirmed robustness, and publication bias was not statistically significant. GRADE assessment indicated low quality of evidence for all-cause mortality, moderate for cardiovascular mortality, and very low quality for remission rate.

**Conclusion:**

Analysis based on a multivariate adjusted model showed that high NLR is an important predictor of poor prognosis in RA patients. As an economical and readily available inflammatory marker, it holds promise for use in RA risk stratification and personalized treatment decision support, but further prospective studies are required to clarify the causal relationship and optimize the clinical cutoff value.

**Systematic review registration:**

https://www.crd.york.ac.uk/prospero/, identifier CRD420251234933,

## 1 Introduction

Rheumatoid arthritis (RA), a persistent autoimmune disease that causes gradual joint deterioration, multi-organ involvement, and a disabling course, has become a major public health problem worldwide (1). This disease not only severely reduces patients’ quality of life but also brings significant additional joint manifestations and complications, with cardiovascular events and infections being the leading causes of death (2). Although the widespread use of biologics and targeted synthetic disease-modifying antirheumatic drugs (DMARDs) has significantly improved joint symptoms and functional outcomes in RA patients, their long-term survival rate remains significantly lower than that of the general population (3, 4). This survival gap underscores the urgency of finding effective prognostic prediction tools. In the complex pathophysiological network of RA, chronic inflammatory immune activation plays a central role (5). It not only drives synovial hyperplasia and bone erosion but also mediates a systemic inflammatory state through a network of pro-inflammatory cytokines. This systemic inflammatory reaction may be strongly associated with poor patient prognosis. In recent years, hematological biomarkers have received increasing attention in the field of disease prognostic assessment due to their simplicity, cost-effectiveness, and reproducibility (6). The NLR, serving as an integrated marker of systemic inflammation and immune regulation, has demonstrated substantial prognostic value in various chronic inflammatory diseases and tumors (7–9). Neutrophils represent innate immune activation and a pro-inflammatory state, and their elevation is often associated with an exacerbated inflammatory response (10); whereas lymphocytes represent adaptive immunity and immune regulation, and their reduction may indicate immune exhaustion or suppression (11). The NLR integrates information from these two types of immune cells, theoretically providing a more comprehensive characterization of the immune-inflammatory imbalance in RA patients. This characteristic may be intrinsically linked to disease activity, treatment response, and even long-term survival outcomes.

Although several studies have explored the relationship between NLR and the prognosis of RA, the existing evidence is significantly inconsistent (12–16). Some studies report a significant link between high NLR and increased all-cause mortality and cardiovascular mortality, while others have not found this association (15, 17–19); the association between NLR and disease remission rate is also controversial. This inconsistency may stem from factors such as heterogeneity in study design, sample size limitations, differences in population characteristics, and inconsistencies in the time points and cutoff values for NLR measurement. More importantly, previous studies have mostly focused on single outcome indicators, lacking a comprehensive assessment of NLR across multiple prognostic dimensions of RA (including mortality, cardiovascular events, and disease remission).

Currently, there is a lack of systematic integration and quantitative analysis of the prognostic value of NLR in multiple dimensions of mortality and disease control outcomes in RA patients. Therefore, a comprehensive assessment of the relationship between NLR and all-cause mortality, cardiovascular mortality, and disease remission rate in RA patients through rigorous meta-analysis has significant theoretical and clinical value. The aim of this study is to conduct a new systematic review and meta-analysis to simultaneously and comprehensively assess the association between NLR and all-cause mortality, cardiovascular mortality, and disease remission rate in RA patients for the first time, in order to provide the most reliable evidence synthesis to clarify existing conflicting evidence and comprehensively assess its clinical prognostic value.

## 2 Methods

### 2.1 Literature search

Following the PRISMA 2020 statement (20), this study was registered in PROSPERO (CRD420251234933). PubMed, Embase, Web of Science, and Cochrane were systematically searched from inception to the present, with the last search update conducted in September 2025, to evaluate the association between NLR and the prognosis of individuals with RA. This study did not apply language restrictions, but did not include grey literature. Search terms were as follows: “Neutrophils”, “Lymphocytes”, “Ratio”, and “RA”. The search query for the PubMed database is detailed below: ((((“Neutrophils”[Mesh]) OR (((((Neutrophil) OR (Polymorphonuclear Neutrophil)) OR (Polymorphonuclear Leukocyte)) OR (LE Cell)) OR (Neutrophil Band Cell))) AND ((“Lymphocytes”[Mesh]) OR ((Lymphocyte) OR (Lymphoid Cell)))) AND (Ratio)) AND [(“Arthritis, Rheumatoid”[Mesh]) OR (RA)]. Additionally, the reference lists of all included studies were scrutinized. Two researchers independently retrieved and evaluated pertinent articles. Any discrepancies in literature identification were addressed through deliberation. Search details are shown in **Table 1**.

**Table 1:** Characteristics and quality assessment of included studies.

Author	Region	Study design	No. of patients	Gender		Mean/median age	NLR cut-off	NOS score
				Male	Female			
Chandrashekara 2020	India	Cohort	72	11	61	44.55	4	7
Dechanuwong 2021	Thailand	Case-control	325	38	287	55	2.6	8
Duan 2024	China	Cohort	153	116	37	71.56	5.27	7
Liu 2025a	China	Cohort	1314	NA	NA	NA	1.64	7
Liu 2025b	China	Cohort	1314	NA	NA	NA	2.47	7
Song 2022	Korea	Cohort	613	0	613	61.9	NA	7
Zhou 2023	China	Cohort	2002	829	1173	56.61	3.28	7
Zhou 2025a	China	Cohort	1683	697	986	57	2.96	7
Zhou 2025b	China	Cohort	1683	697	986	57	2.96	7

### 2.2 Inclusion and exclusion criteria

Inclusion criteria:

P: patients diagnosed with RA.
E: high NLR.
C: low NLR.
O: any clinical outcome of RA, including all-cause mortality, cardiovascular mortality, and remission, etc.
S: study designs were cohort or case-control.

Protocols, reviews, and other non-original publications—including letters, comments, replies, abstracts, and corrections—were excluded. In addition, unpublished studies, single-arm designs, and studies lacking sufficient data (i.e., those from which the odds ratio (OR) and 95% confidence intervals (CIs) could not be extracted or derived) were also excluded from the analysis.

### 2.3 Data abstraction

The two researchers independently extracted the data using Microsoft Excel. After completion, they verified the results. For any data items with discrepancies, both researchers first reviewed the relevant sections of the original literature and discussed them. If they still could not reach an agreement, the data was submitted to a third researcher for review and arbitration, with the third party’s judgment becoming the final decision. This process ensured the accuracy and consistency of all extracted data. The following information was abstracted: first author name, year of publication, study design, region, population, sample size, gender, age, NLR cut-off, the OR and 95% CI of all-cause mortality, cardiovascular mortality, and remission, etc. OR values were preferentially extracted from multivariate analysis models.

### 2.4 Quality evaluation

Newcastle-Ottawa Scale (NOS) was used to assess study quality (21), with studies scoring 7–9 points regarded as high quality (22). Two authors independently assessed all included studies’ quality, with disagreements settled by discussion.

### 2.5 Statistical analysis

Review Manager 5.4.1 was applied for all analyses. OR with 95% CIs were generated using a random-effects model (DerSimonian–Laird). Heterogeneity for each outcome was quantified using the Chi-squared (χ²) test (Cochran’s Q) and the inconsistency index (I²) (23, 24). A *P* value < 0.1 or *I*^2^ > 50% indicated high heterogeneity. For results with >2 included studies, sensitivity analysis assessed each included study’s influence on the total metric. Assessment of publication bias was conducted using funnel plots together with Egger’s regression test (25). Values of P < 0.05 were taken as evidence of significant bias. For results with publication bias, the impact of bias on the results was assessed using the trim-and-fill method. In addition, evidence for all outcomes was evaluated using GRADE and categorized into high, moderate, low, or very low quality (26). Due to insufficient research volume, we were unable to perform subgroup analysis and meta-regression.

## 3 Results

### 3.1 Literature retrieval and study characteristics

**Figure 1:**
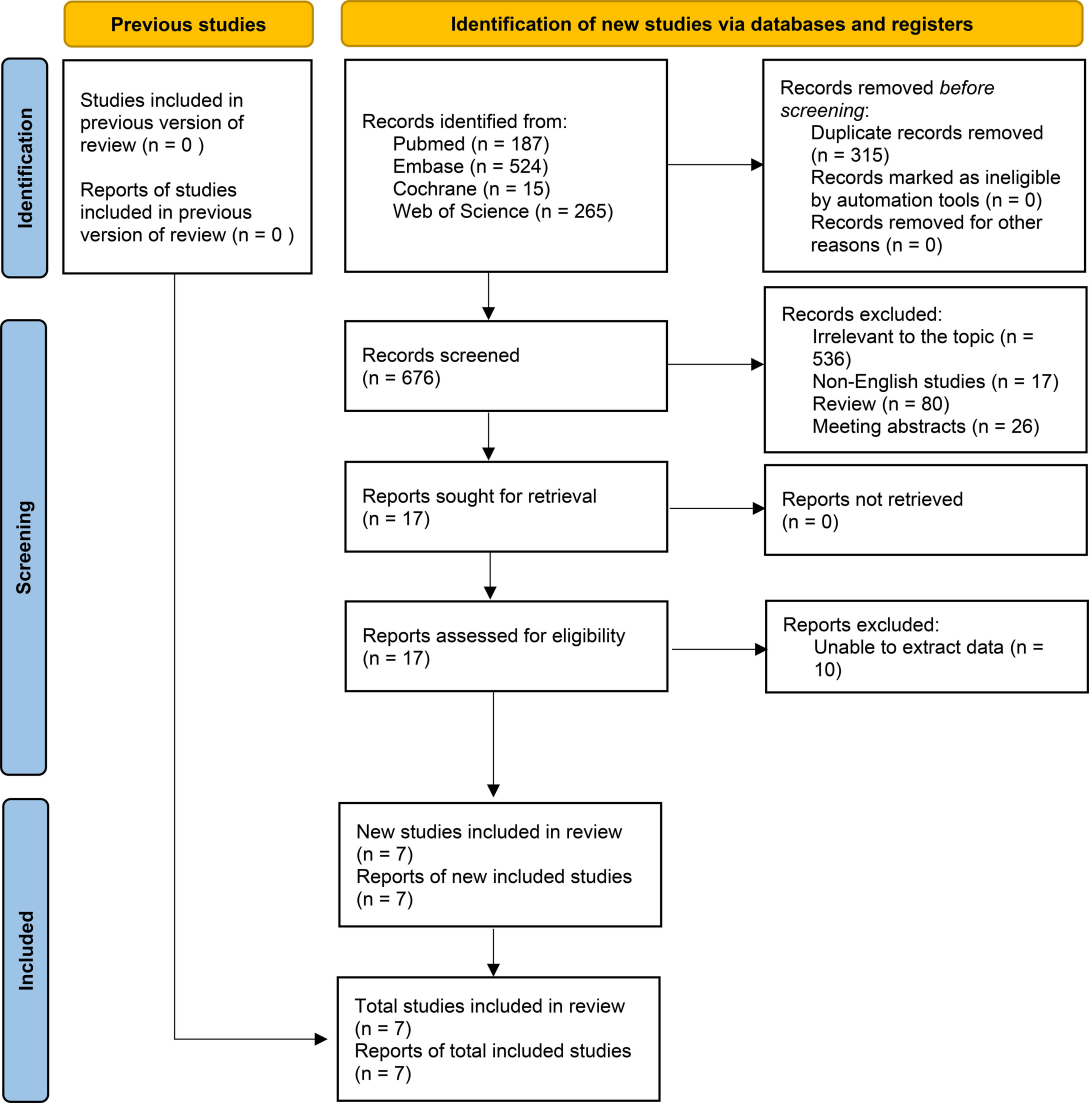
Flowchart of the systematic search and selection process.

The flowchart illustrating the literature search and selection process is presented in **Figure 1**. A total of 991 studies were identified in PubMed (n = 187), Embase (n = 524), Web of Science (n = 265), and Cochrane (n = 15) included. Once duplicates were excluded, 676 titles and abstracts were left for assessment. Ultimately, 7 studies were included for analysis (15–19, 27, 28). **Table 1** presents each eligible study’s characteristics and quality assessment. The included literature was published between 2020-2025.

A meta-analysis of seven observational studies consistently demonstrated that higher baseline NLR levels were associated with a significantly increased risk of all-cause mortality and cardiovascular mortality in RA patients, as well as a significantly reduced disease remission rate. Specific results are as follows:

### 3.2 Association between NLR and all-cause mortality

**Figure 2:**
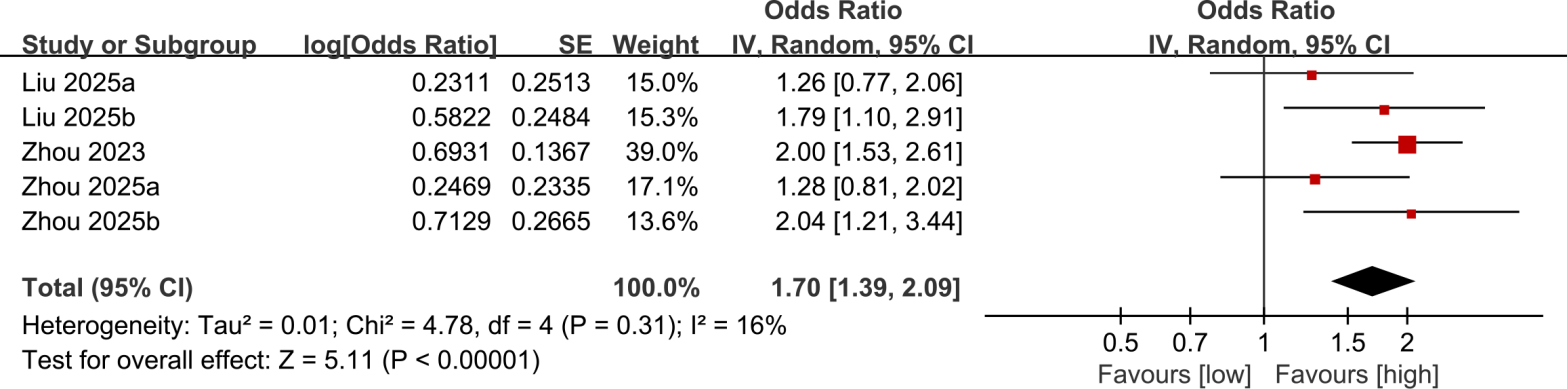
Forest plots of the association between NLR and all-cause mortality.

Five studies were synthesized for the relationship between NLR and all-cause mortality. Meta-analysis revealed that RA patients with higher NLR had significantly higher all-cause mortality (OR: 1.70; 95% CI: 1.39, 2.09; *P* <0.00001), without significant heterogeneity (*I*^2^ = 16%, *P* = 0.31) (**Figure 2**).

### 3.3 Association between NLR and cardiovascular mortality

**Figure 3:**
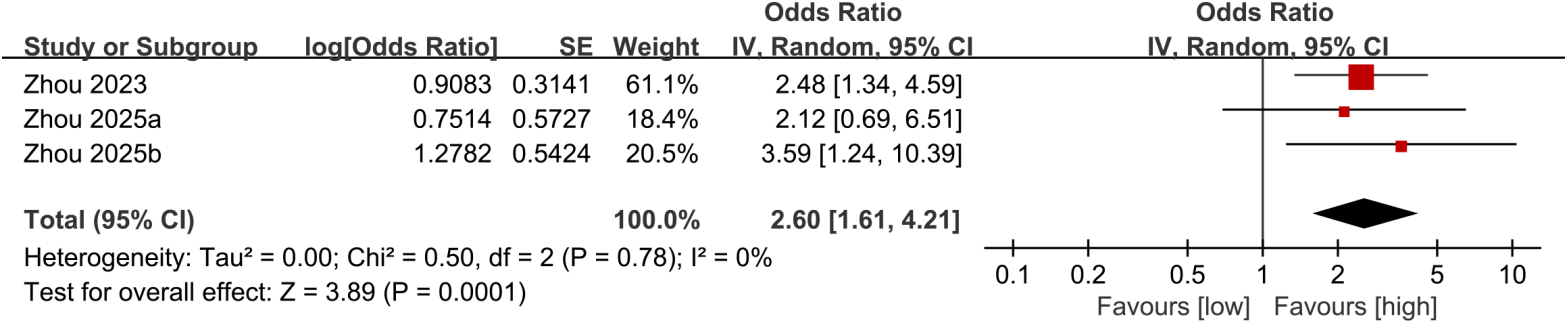
Forest plots of the association between NLR and cardiovascular mortality.

Three studies were synthesized for the relationship between NLR and cardiovascular mortality. Meta-analysis revealed that RA patients with higher NLR had significantly higher cardiovascular mortality (OR: 2.60; 95% CI: 1.61, 4.21; *P* = 0.0001), without significant heterogeneity (*I*^2^ = 0%, *P* = 0.78) (**Figure 3**).

### 3.4 Association between NLR and remission

**Figure 4:**
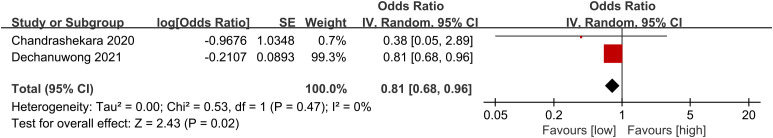
Forest plots of the association between NLR and remission rate.

Two studies were synthesized for the association between NLR and remission. Meta-analysis revealed that RA patients with higher NLR had significantly lower remission rates (OR: 0.81; 95% CI: 0.68, 0.96; *P* = 0.02), without significant heterogeneity (*I*^2^ = 0%, *P* = 0.47) (**Figure 4**).

### 3.5 Publication bias

**Figure 5:**
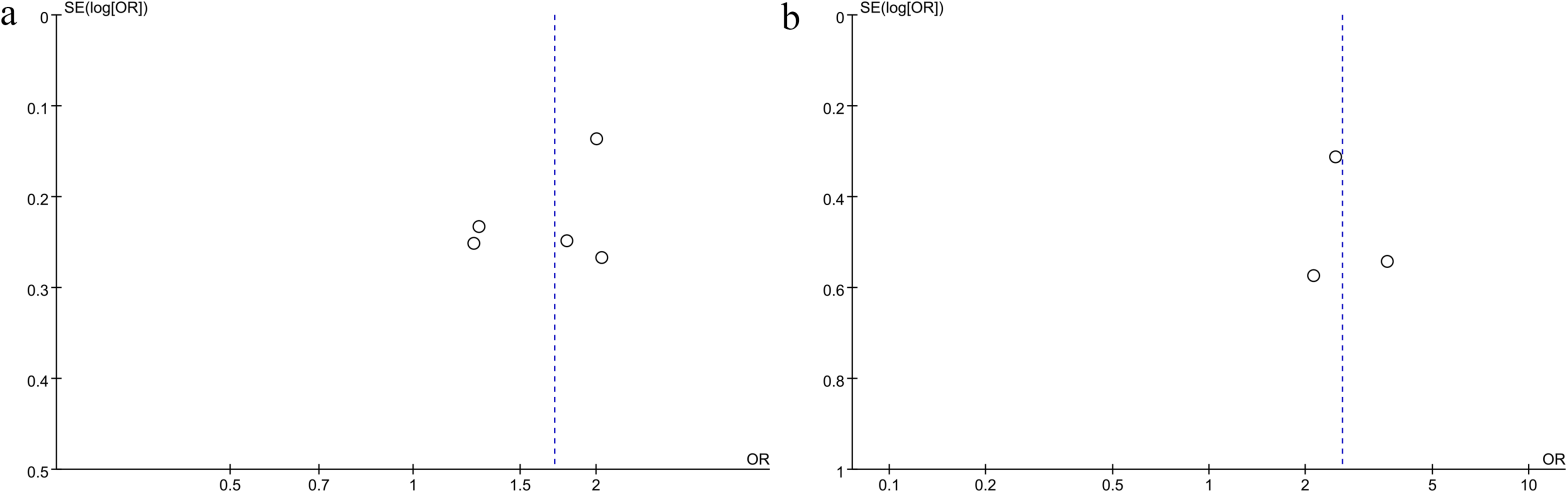
Funnel plots of the association between NLR and **(A)** all-cause mortality and **(B)** cardiovascular mortality.

The potential publication bias related to the association between NLR and all-cause or cardiovascular mortality was examined using funnel plots and Egger’s regression tests. Results from Egger’s test, along with the symmetrical appearance of the funnel plots, showed no significant publication bias for all-cause mortality (Egger’s P = 0.325; **Figure 5**) or for cardiovascular mortality (Egger’s P = 0.831; **Figure 5**).

### 3.6 Sensitivity analysis

**Figure 6:**
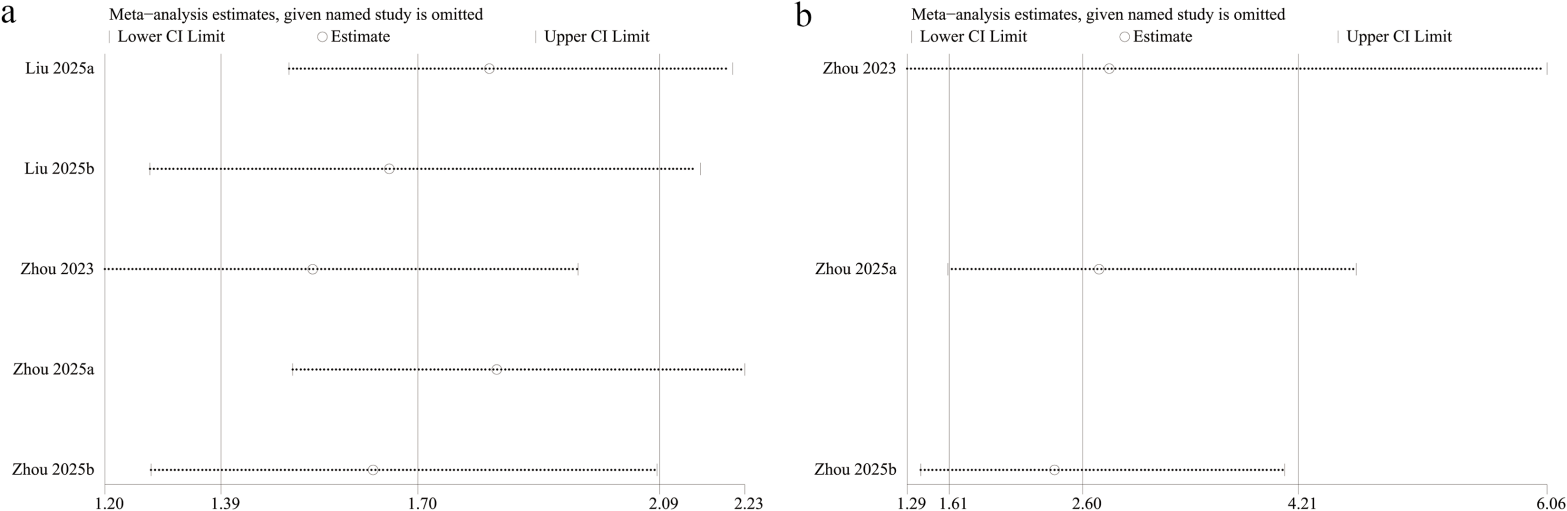
Sensitivity analysis of the association between NLR and **(A)** all-cause mortality and **(B)** cardiovascular mortality.

To assess the robustness of the findings, a sensitivity analysis was conducted by sequentially removing each included study and recalculating the overall OR for the association between NLR and all-cause and cardiovascular mortality. The recalculated ORs remained consistent across all iterations for both all-cause mortality (**Figure 6**) and cardiovascular mortality (**Figure 6**), indicating stable results.

### 3.7 GRADE rating

Regarding the evidence recommendations, all-cause mortality was rated as low-quality evidence due to the observational study design and the lack of escalation and de-escalation factors. Cardiovascular mortality was upgraded to moderate-quality evidence due to its magnitude of effect (according to the official guidelines of the GRADE working group, when the point estimate of OR is >2 (or <0.5) and the confidence interval is narrow, indicating a strong association between the intervention or exposure factor and the outcome, the quality of evidence can be upgraded by one level due to the “magnitude of effect “). However, the remission rate was rated as very low-quality evidence due to significant imprecision. Detailed GRADE classification results are shown in **Table 2**.

**Table 2:** Grade rating of each outcome.

No. of studies	Outcomes	OR	95%CI	*I*^2^; P value	Risk of bias	Inconsistency	Indirectness	Imprecision	Publication bias	Plausible confounding	Magnitude of effect	Dose-response gradient	Grade
5	All-cause mortality	1.70	1.39, 2.09	16%; P = 0.31	No serious risk	No serious inconsistency	No serious indirectness	No serious imprecision	Undetected	Would not reduce effect	No	No	Low
3	Cardiovascular mortality	2.60	1.61, 4.21	0%; P = 0.78	No serious risk	No serious inconsistency	No serious indirectness	No serious imprecision	Undetected	Would not reduce effect	Yes	No	Moderate
2	Remission	0.81	0.68, 0.96	0%; P = 0.47	No serious risk	No serious inconsistency	No serious indirectness	Serious imprecision	NA	Would not reduce effect	No	No	Very low

## 4 Discussion

This meta-analysis of seven studies confirmed that high NLR was significantly associated with increased all-cause mortality and cardiovascular mortality in patients with RA, and was also associated with decreased disease remission rate, with these associations being highly statistically significant (P<0.05). Notably, no significant heterogeneity was found in any of the analyses (I²=0-16%), and sensitivity analysis showed robust results, and publication bias assessment indicated no significant bias. These results indicate that NLR may serve as a reliable predictor of RA prognosis, with value extending beyond mortality prediction to the assessment of disease activity. It is crucial to interpret these findings within the inherent constraints of the observational study designs from which the data were derived. While our meta-analysis synthesizes adjusted estimates and demonstrates significant associations, the non-randomized nature of all included studies precludes the establishment of causal relationships between elevated NLR and the examined outcomes. Unmeasured or residual confounding may persist despite statistical adjustments in the original studies, potentially influencing the observed effect sizes. Therefore, the results should be viewed as providing robust evidence for a consistent prognostic association, rather than definitive proof of causality. This fundamental methodological consideration underpins the need for cautious interpretation and highlights the necessity for future prospective validation.

From a biological perspective, the association between NLR and poor prognosis in RA may be mediated through multiple pathways: elevated neutrophils reflect a persistent state of systemic inflammation, and the proteases, reactive oxygen species, and inflammatory mediators (such as IL-1β and TNF-α) released by neutrophils not only exacerbate joint damage but may also promote the formation and instability of atherosclerotic plaques, increasing the risk of cardiovascular events (29–31); while lymphopenia may indicate impaired immune regulation and immunosenescence, which is associated with increased susceptibility to infection and poor treatment response (32, 33).

Compared to previous studies, our findings are consistent with those reporting the prognostic value of NLR, but provide more comprehensive and precise evidence. The strong relationship between NLR and cardiovascular mortality is particularly significant, as cardiovascular disease is a leading cause of excess mortality in RA patients (34, 35). Our meta-analysis quantified the strength of this association, providing strong evidence for NLR as a cardiovascular risk stratification tool. Furthermore, this study is the first to confirm a negative correlation between NLR and disease remission rate through meta-analysis, expanding our understanding of the clinical value of NLR and suggesting it may simultaneously reflect disease activity and long-term risk. Compared to studies that did not find significant associations, our increased sample size and more rigorous statistical methods may have improved the ability to detect the true effect. It should be noted that the effect size found in this study is comparable to reports in other inflammatory diseases (such as cardiovascular disease and cancer) (36–38), suggesting that the prognostic value of NLR may have cross-disease generalizability, but its specific mechanisms in RA still require further investigation.

From a clinical application perspective, the findings of this study have significant practical implications. NLR, as a simple, economical indicator derived from routine blood tests, is well-suited for widespread application in resource-constrained environments. Clinicians can use NLR for risk stratification: RA patients with elevated NLR should be considered high-risk, requiring enhanced management of cardiovascular risk factors, optimized disease activity control, and closer follow-up monitoring. In treatment strategy development, NLR may help identify patient groups requiring more aggressive anti-inflammatory treatment or cardiovascular prevention measures. Furthermore, dynamic changes in NLR may serve as a monitoring indicator of treatment response, and its trend may be more predictive than a single measurement (39). However, the clinical application of NLR still faces several challenges, the most important of which is the determination of a standardized and optimized cutoff value. The studies included in this study used different NLR cutoff values; future large-sample studies are needed to determine the optimal cutoff value, and age, sex, and ethnicity-specific criteria may also need to be considered.

The methodological strengths of this study deserve emphasis. We strictly followed the PRISMA guidelines, conducted a comprehensive literature search, and minimized the risk of missing relevant studies. We employed a random-effects model to address potential heterogeneity, and validated the robustness of the results through sensitivity analysis. Publication bias assessment showed no significant bias, enhancing the credibility of our conclusions. Of particular importance, we used the GRADE system to grade the quality of evidence, with evidence for all-cause mortality and cardiovascular mortality rated as low and moderate quality, respectively, providing transparency for clinical decision-making. However, certain limitations of this study must be recognized. First, all included studies were observational in design, making it impossible to establish causal relationships, and residual confounding factors (such as disease activity, treatment modality, and comorbidities) could affect the results. Second, the sample size was relatively limited, particularly the response rate analysis which included only two studies, limiting the potential for statistical power and subgroup analysis. Third, differences existed among studies in population characteristics, NLR measurement time, and cutoff values; although no significant heterogeneity was detected, these differences could affect the precision of the results. Finally, most studies were from specific regions, which may limit the general applicability of the results.

Considering the results and limitations of this study, future work should move in several key directions. To begin with, large, multi-center prospective cohort studies are required to confirm the prognostic significance of NLR and to determine a standardized cutoff value. Second, the combined value of NLR with other known prognostic factors (such as disease activity indices, radiographic progression, and autoantibody status) should be explored to construct a comprehensive predictive model. Third, studying the dynamic changes in NLR and their association with prognosis may provide more valuable clinical information. From a mechanistic perspective, experimental studies are essential to clarify the specific links between NLR and the pathophysiological processes of RA, particularly its mediating role in the development of cardiovascular complications. Finally, interventional studies are worth conducting to evaluate whether treatment strategies aimed at reducing NLR can improve patient outcomes.

## 5 Conclusion

In conclusion, this study confirms that NLR, an inflammatory marker derived from routine blood tests, is low-cost, and easily accessible, and is closely related to the long-term survival and disease activity of RA patients. This provides evidence-based medical support for its use as a practical adjunct tool for risk stratification, prognostic assessment, and individualized management decision support in RA patients. Future research needs to clarify the causal role of NLR through prospective designs, establish population-specific cutoff values, and explore its synergistic value in combination with emerging biomarkers to promote the optimization of precision prognostic management strategies for RA.

## Data Availability

The original contributions presented in the study are included in the article/**Supplementary Material**. Further inquiries can be directed to the corresponding author.

## References

[1] Zhang Z Liu Y Liang X Wang Q Xu M Yang X . Advances in nanodelivery systems based on apoptosis strategies for enhanced rheumatoid arthritis therapy. Acta Biomater. (2025) 197:87–103. doi: 10.1016/j.actbio.2025.03.043. PMID: 40154765

[2] Li H Fu S Shen P Zhang X Yang Y Guo J . Mitochondrial pathways in rheumatoid arthritis: therapeutic roles of traditional Chinese medicine and natural products. Phytomedicine. (2025) 146:157106. doi: 10.1016/j.phymed.2025.157106. PMID: 40782763

[3] Bazzazzadehgan S . Comparative effectiveness of biologic or targeted synthetic disease-modifying antirheumatic drugs in terms of opioid tapering and discontinuation among medicare beneficiaries with rheumatoid arthritis (Electronic Theses and Dissertations). (2025). Available online at: https://egrove.olemiss.edu/etd/3243.

[4] Jain A Bishnoi M Prajapati SK Acharya S Kapre S Singhvi G . Targeting rheumatoid arthritis: a molecular perspective on biologic therapies and clinical progress. J Biol Eng. (2025) 19:67. doi: 10.1186/s13036-025-00534-8. PMID: 40707975 PMC12291358

[5] Liao H Zheng J Lu J Shen H . NF-κB signaling pathway in rheumatoid arthritis: mechanisms and therapeutic potential. Mol Neurobiol. (2025) 62:6998–7021. doi: 10.1007/s12035-024-04634-2. PMID: 39560902

[6] Sah AK . The potential of cancer biomarkers. Amsterdam, Netherlands: Elsevier (2025). p. 211–38.

[7] Chmielewski PP Strzelec B Mozdziak P Kempisty B . Neutrophil-to-lymphocyte ratio as a prognostic biomarker for long-term survival in older adults at a mental health care center: A historical cohort analysis. J Clin Med. (2025) 14:2509. doi: 10.3390/jcm14072509. PMID: 40217958 PMC11989978

[8] Lagunas-Rangel FA . Neutrophil-to-lymphocyte ratio in aging: Trends and clinical implications. Exp Gerontology. (2025) 211:112908. doi: 10.1016/j.exger.2025.112908. PMID: 40992643

[9] Liu Y Wang R Yuan J Zhao J . The role of neutrophil-to-lymphocyte ratio in the prognosis of chronic kidney disease: insights from the NHANES cohort study. Front Syst Biol. (2025) 5:1656683. doi: 10.3389/fsysb.2025.1656683. PMID: 41220423 PMC12597963

[10] Yin S Zhuo N Duan Q Song J Yang C . Single-cell sequencing reveals that neutrophils mediate the inflammatory response in the placenta of women with GDM. J Mol Histol. (2026) 57(1):50. doi: 10.1007/s10735-025-10707-w, PMID: 41546731

[11] Han L Wu T Zhang Q Qi A Zhou X . Immune tolerance regulation is critical to immune homeostasis. J Immunol Res. (2025) 2025:5006201. doi: 10.1155/jimr/5006201. PMID: 39950084 PMC11824399

[12] Pan YJ Su KY Shen CL Wu YF . Correlation of hematological indices and acute-phase reactants in rheumatoid arthritis patients on disease-modifying antirheumatic drugs: A retrospective cohort analysis. J Clin Med. (2023) 12(24):7611. doi: 10.3390/jcm12247611. PMID: 38137680 PMC10744259

[13] Nagai Y Yokogawa N Shimada K Sugii S . Utility of the neutrophil-to-lymphocyte ratio for predicting bacterial infection in patients with rheumatoid arthritis receiving Tocilizumab. Rheumatol Int. (2020) 40:2039–46. doi: 10.1007/s00296-020-04705-2. PMID: 32965587

[14] Boulos D Proudman SM Metcalf RG McWilliams L Hall C Wicks IP . The neutrophil-lymphocyte ratio in early rheumatoid arthritis and its ability to predict subsequent failure of triple therapy. Semin Arthritis Rheumatism. (2019) 49:373–6. doi: 10.1016/j.semarthrit.2019.05.008. PMID: 31248587

[15] Zhou F Xie Y Zhang Y Jiang P . Correlation of Naples prognostic score with the risk of all-cause and cardiovascular mortality in individuals with rheumatoid arthritis: A cross-sectional analysis of the NHANES 2001-2018. Joint Bone Spine. (2025) 92:105928. doi: 10.1016/j.jbspin.2025.105928. PMID: 40436104

[16] Liu Y Liu Y Fan S Yang J Xu M Zhao L . Correlation between CBC-derived inflammatory indicators and all-cause mortality with rheumatoid arthritis: a population-based study. Front Med. (2025) 12:1538710. doi: 10.3389/fmed.2025.1538710. PMID: 40557044 PMC12185416

[17] Zhou E Wu J Zhou X Yin Y . The neutrophil-lymphocyte ratio predicts all-cause and cardiovascular mortality among U.S. adults with rheumatoid arthritis: results from NHANES 1999-2020. Front Immunol. (2023) 14:1309835. doi: 10.3389/fimmu.2023.1309835. PMID: 38045692 PMC10690944

[18] Song BW Kim AR Moon DH Kim YK Kim GT Ahn EY . Associations of neutrophil-to-lymphocyte ratio, platelet-to-lymphocyte ratio and monocyte-to-lymphocyte ratio with osteoporosis and incident vertebral fracture in postmenopausal women with rheumatoid arthritis: A single-center retrospective cohort study. Med (Kaunas Lithuania). (2022) 58(7):852. doi: 10.3390/medicina58070852. PMID: 35888571 PMC9321011

[19] Dechanuwong P Phuan-Udom R . Hematological parameters as a predictor of disease remission in patients with rheumatoid arthritis. Ann Med Surg (2012). (2021) 72:103085. doi: 10.1016/j.amsu.2021.103085. PMID: 34868575 PMC8626573

[20] Page MJ McKenzie JE Bossuyt PM Boutron I Hoffmann TC Mulrow CD . The PRISMA 2020 statement: an updated guideline for reporting systematic reviews. BMJ (Clinical Res Ed). (2021) 372:n71. doi: 10.31222/osf.io/v7gm2. PMID: 33782057 PMC8005924

[21] Wells G Shea B O’Connell D Peterson J Welch V Losos M . The Newcastle-Ottawa Scale (NOS) for assessing the quality of nonrandomised studies in meta-analyses (2011). Available online at: http://www.ohri.ca/programs/clinical_epidemiology/oxford.asp (Accessed September 1, 2025).

[22] Kim SR Kim K Lee SA Kwon SO Lee JK Keum N . Effect of red, processed, and white meat consumption on the risk of gastric cancer: an overall and dose−Response meta-analysis. Nutrients. (2019) 11(4):826. doi: 10.3390/nu11040826. PMID: 30979076 PMC6520977

[23] Higgins JP Thompson SG . Quantifying heterogeneity in a meta-analysis. Stat Med. (2002) 21:1539–58. doi: 10.1191/026921699666884611. PMID: 12111919

[24] Li S Qu Z Li Y Ma X . Efficacy of e-health interventions for smoking cessation management in smokers: a systematic review and meta-analysis. EClinicalMedicine. (2024) 68:102412. doi: 10.1016/j.eclinm.2023.102412. PMID: 38273889 PMC10809126

[25] Egger M Davey Smith G Schneider M Minder C . Bias in meta-analysis detected by a simple, graphical test. BMJ (Clinical Res Ed). (1997) 315:629–34. doi: 10.1136/bmj.315.7109.629. PMID: 9310563 PMC2127453

[26] Guyatt G Oxman AD Akl EA Kunz R Vist G Brozek J . GRADE guidelines: 1. Introduction-GRADE evidence profiles and summary of findings tables. J Clin Epidemiol. (2011) 64:383–94. doi: 10.1016/j.jclinepi.2010.04.026. PMID: 21195583

[27] Chandrashekara S Lingaraju DC Renuka P Anupama KR . Potential of neutrophil to lymphocyte ratio in predicting sustained remission in rheumatoid arthritis compared to other immune activation markers. Indian J Med Res. (2020) 152:234–43. doi: 10.4103/ijmr.ijmr_1676_18. PMID: 33107483 PMC7881809

[28] Duan R Lin L Zou Y Lin X . Neutrophil-to-lymphocyte ratio combined with albumin to globulin ratio for predicting rheumatoid arthritis-associated pneumonia. Am J Trans Res. (2024) 16:6796–803. doi: 10.62347/jpnv8527. PMID: 39678609 PMC11645621

[29] Wang Y Shu Y Wu H . Advances in drug research targeting neutrophils for the treatment of rheumatoid arthritis. Curr Rheumatol Rep. (2025) 27:1–11. doi: 10.1007/s11926-025-01201-z. PMID: 40928697

[30] Deng Y Li J Wu R . Neutrophils in rheumatoid arthritis synovium: implications on disease activity and inflammation state. J Inflamm Res. (2025) 18:4741–53. doi: 10.2147/jir.s503144. PMID: 40206809 PMC11980796

[31] Bhargav A Kumar V Rai NK . Roles of neutrophils in autoimmune diseases and cancers. Int J Mol Sci. (2025) 26:9040. doi: 10.3390/ijms26189040. PMID: 41009604 PMC12469507

[32] Wen J Liu J Wan L Wang F . New insights into the role of cellular senescence and rheumatic diseases. Front Immunol. (2025) 16:1557402. doi: 10.3389/fimmu.2025.1557402. PMID: 40453080 PMC12122340

[33] Schwarz T Almanzar G Völkl S Feuchtenberger M Leierer J Schmidt C . Diverging effects of tumor necrosis factor inhibitors and conventional synthetic disease-modifying antirheumatic drugs on immunosenescence and inflammageing in rheumatoid arthritis: a cross-sectional analysis. Immun Ageing. (2025) 22:21. doi: 10.1186/s12979-025-00508-w. PMID: 40405219 PMC12096643

[34] Xie X Wei G Tang Z Chen H Lin X Huang C . Investigating the causal relationship between rheumatoid arthritis and cardiovascular disease: a Mendelian randomization study. Clin Rheumatol. (2025) 44:1057–67. doi: 10.1007/s10067-025-07357-4. PMID: 39909965

[35] Ai-Miahee AA Al-Attabi MRS . Cardiovascular risk assessment in patients with rheumatoid arthritis. Int J Environ Sci. (2025) 11:1152–62. doi: 10.1097/md.0000000000006983. PMID: 28885321 PMC6393106

[36] Zhang X Li D Du Y . Prognostic value of the neutrophil to lymphocyte ratio for cardiovascular diseases: research progress. Am J Trans Res. (2025) 17:1170. doi: 10.62347/kvcv7377. PMID: 40092102 PMC11909572

[37] Vida B Keszthelyi E Tóth Z Lőczi L Sebők B Merkely P . The neutrophil-to-lymphocyte ratio (NLR) as a potential predictor in conization outcomes for cervical cancer. Cancers. (2025) 17:1856. doi: 10.3390/cancers17111856. PMID: 40507335 PMC12153780

[38] Chi X Bi Q You L Zhou Y Zhao C . Predictive value of NLR for the occurrence and clinical outcomes of hypertension: a systematic review and meta-analysis. Biomarkers Med. (2025) 19:783–91. doi: 10.1080/17520363.2025.2542111. PMID: 40757455 PMC12416170

[39] Li M Spakowicz D Burkart J Patel S Husain M He K . Change in neutrophil to lymphocyte ratio during immunotherapy treatment is a non-linear predictor of patient outcomes in advanced cancers. J Cancer Res Clin Oncol. (2019) 145:2541–6. doi: 10.1007/s00432-019-02982-4. PMID: 31367835 PMC6751277

