# Supplementary material for: Association between neutrophil-to-lymphocyte ratio and the prognosis of patients with rheumatoid arthritis: a meta-analysis
Source: Front Immunol. 2026 Apr 1;17:1770565. doi: 10.3389/fimmu.2026.1770565 (PMC13079348; doi:10.3389/fimmu.2026.1770565)
Supplement: Supplementary file 1 [file DataSheet1.docx]

**Supplementary Materials**

TableS1 Literature search strategy

Pubmed-187

(((("Neutrophils"[Mesh]) OR (((((Neutrophil) OR (Polymorphonuclear Neutrophil)) OR (Polymorphonuclear Leukocyte)) OR (LE Cell)) OR (Neutrophil Band Cell))) AND (("Lymphocytes"[Mesh]) OR ((Lymphocyte) OR (Lymphoid Cell)))) AND (Ratio)) AND (("Arthritis, Rheumatoid"[Mesh]) OR (Rheumatoid Arthritis))

Embase-524

((Neutrophils or (Neutrophil or Polymorphonuclear Neutrophil or Polymorphonuclear Leukocyte or LE Cell or Neutrophil Band Cell)) and (Lymphocytes or (Lymphocyte or Lymphoid Cell)) and Ratio and (Arthritis, Rheumatoid or Rheumatoid Arthritis)).af.

Cochrane-15

((Neutrophils or (Neutrophil or Polymorphonuclear Neutrophil or Polymorphonuclear Leukocyte or LE Cell or Neutrophil Band Cell)) and (Lymphocytes or (Lymphocyte or Lymphoid Cell)) and Ratio and (Arthritis, Rheumatoid or Rheumatoid Arthritis)).af.

Web of Science-265

((((Neutrophils) OR (((((Neutrophil) OR (Polymorphonuclear Neutrophil)) OR (Polymorphonuclear Leukocyte)) OR (LE Cell)) OR (Neutrophil Band Cell))) AND ((Lymphocytes) OR ((Lymphocyte) OR (Lymphoid Cell)))) AND (Ratio)) AND ((Arthritis, Rheumatoid) OR (Rheumatoid Arthritis)) (Topic)
